# Angiotensin AT_1_ and AT_2_ receptor heteromer expression in the hemilesioned rat model of Parkinson’s disease that increases with levodopa-induced dyskinesia

**DOI:** 10.1186/s12974-020-01908-z

**Published:** 2020-08-17

**Authors:** Rafael Rivas-Santisteban, Ana I. Rodriguez-Perez, Ana Muñoz, Irene Reyes-Resina, José Luis Labandeira-García, Gemma Navarro, Rafael Franco

**Affiliations:** 1 Department of Biochemistry and Molecular Biomedicine, School of Biology, Universitat de Barcelona, Barcelona, Spain; 2 Centro de Investigación en Red, enfermedades Neurodegenerativas, CiberNed, Instituto de Salud Carlos III, Madrid, Spain; 3 Laboratory of Cellular and Molecular Neurobiology of Parkinson’s disease, Research Center for Molecular Medicine and Chronic Diseases (CIMUS), Department of Morphological Sciences, IDIS, University of Santiago de Compostela, Santiago de Compostela, Spain; 4 Current adress: RG Neuroplasticity, Leibniz Institute for Neurobiology, 39118 Magdeburg, Germany; 5 Department of Biochemistry and Physiology, School of Pharmacy and Food Sciences, Universitat de Barcelona, Barcelona, Spain; 6 School of Chemistry, Universitat de Barcelona, Barcelona, Spain

**Keywords:** Neuroinflammation, Heteromer, G-protein-coupled receptor (GPCR), Dyskinesia, Levodopa

## Abstract

**Background/aims:**

The renin-angiotensin system (RAS) is altered in Parkinson’s disease (PD), a disease due to substantia nigra neurodegeneration and whose dopamine-replacement therapy, using the precursor levodopa, leads to dyskinesias as the main side effect. Angiotensin AT_1_ and AT_2_ receptors, mainly known for their role in regulating water homeostasis and blood pressure and able to form heterodimers (AT_1/2_Hets), are present in the central nervous system. We assessed the functionality and expression of AT_1/2_Hets in Parkinson disease (PD).

**Methods:**

Immunocytochemistry was used to analyze the colocalization between angiotensin receptors; bioluminescence resonance energy transfer was used to detect AT_1/2_Hets. Calcium and cAMP determination, MAPK activation, and label-free assays were performed to characterize signaling in homologous and heterologous systems. Proximity ligation assays were used to quantify receptor expression in mouse primary cultures and in rat striatal sections.

**Results:**

We confirmed that AT_1_ and AT_2_ receptors form AT_1/2_Hets that are expressed in cells of the central nervous system. AT_1/2_Hets are novel functional units with particular signaling properties. Importantly, the coactivation of the two receptors in the heteromer reduces the signaling output of angiotensin. Remarkably, AT_1/2_Hets that are expressed in both striatal neurons and microglia make possible that candesartan, the antagonist of AT_1_, increases the effect of AT_2_ receptor agonists. In addition, the level of striatal expression increased in the unilateral 6-OH-dopamine lesioned rat PD model and was markedly higher in parkinsonian-like animals that did not become dyskinetic upon levodopa chronic administration if compared with expression in those that became dyskinetic.

**Conclusion:**

The results indicate that boosting the action of neuroprotective AT_2_ receptors using an AT_1_ receptor antagonist constitutes a promising therapeutic strategy in PD.

## Introduction

The renin angiotensin system (RAS) is composed of enzymes that produce angiotensin (Ang) peptides and of cell surface receptors that convey cytocrin signals to achieve specific cell responses. There are two angiotensin receptors (AT_1_R and AT_2_R) that belong to the superfamily of G-protein-coupled receptors. RAS has been abundantly studied in the periphery, mainly in relation with the control of arterial blood pressure. However, different laboratories have provided solid evidence of the relevant role of RAS in the central nervous system (CNS). Ang is an important regulator of motor control, and AT_1_R and AT_2_R have been suggested as targets to combat Parkinson’s disease (PD) and related conditions such as levodopa (L-DOPA)-induced dyskinesias [38, 45].

Age is a main risk factor for sporadic PD, which is characterized by dysregulation of the dopaminergic function due to the death of dopaminergic neurons of the *substantia nigra* (SN). A local RAS has been reported in the SN [20, 61], in which overactivity of AT_1_R correlates with aging-related alterations, neuronal death [22, 31], and neuroinflammation (Labandeira-Garcia et al., [29, 30, 52]). Microglial cells are the main mediators of neuroinflammation and despite once activated they are considered as detrimental, it is now known that they may undertake the pro-inflammatory (M1) or the neuroprotective (M2) phenotype. The search for pharmacological tools targeting G-protein-coupled receptors to convert M1 into M2 phenotype is an active field of research. The role of AT_2_R and the interplay between the two receptors in the above-mentioned changes due to Ang action in the aged or in the pathological brain is still unclear.

The cognate proteins for coupling to AT_1_R and AT_2_R are, respectively, Gq (also Gi) and Gi. Accordingly, agonists of AT_1_R may mobilize calcium ion from intracellular stores, whereas agonists of AT_2_R decrease the activity of adenylyl cyclase thus depressing the cAMP/PKA signaling (https://www.guidetopharmacology.org). Interestingly, the two receptors may interact, leading to the formation of receptor heteromers with particular properties: pharmacological, functional, or both [15, 48]. On the one hand, heteromerization modifies receptor trafficking and ß-arrestin recruitment [48]. On the other hand, Ang II induces the formation of heteromers of the two receptors (AT_1/2_Hets) in luminal membranes of kidney tubular epithelial LLC-PK1 cells. In these cells, the peptide activates a calcium channel, sarco/endoplasmic reticulum Ca^2+^-ATPase (SERCA), that in kidney cells participates in the control of blood pressure [14].

The main target for pharmacological anti-parkinsonian interventions is the striatum that receives the SN dopaminergic input needed for motor control. What is important is to know whether AT_1_ and AT_2_ receptors interact in the CNS, which is their physiological function and how their expression alters the course of a neurodegenerative disease. Accordingly, the aims of this paper were to (i) get further insight into the properties of AT_1/2_Hets in a heterologous expression system; (ii) investigate the expression and function of AT_1_R, AT_2_R, and AT_1/2_Hets in striatal neurons; and (iii) investigate the expression and function of AT_1_R, AT_2_R, and AT_1/2_Hets in striatal microglia in resting and activated states. The results show that AT_2_R are expressed in neurons and in activated microglia where they interact with AT_1_R to form AT_1/2_Hets. Accordingly, a final aim was to discover differential expression of AT_1/2_Hets in striatal samples from parkinsonian and dyskinetic animals.

## Materials and methods

### Reagents

Lipopolysaccharide (LPS), interferon-γ (IFN-γ), and forskolin, Angiotensin II (Ang II), CGP-42112A (CGP), Candesartan (CAN) and PD123319 (PD) were purchased from Sigma-Aldrich (St Louis, MO).

### HEK-293T cells and primary cultures

Human embryonic kidney (HEK-293T) cells were grown in Dulbecco’s modified Eagle’s medium (DMEM) (Gibco) supplemented with 2 mM l-glutamine, 100 μg/mL sodium pyruvate, 100 U/mL penicillin/streptomycin, MEM non-essential amino acids solution (1/100), and 5% (v/v) heat-inactivated fetal bovine serum (FBS) (all supplements were from Invitrogen, Paisley, Scotland, UK).

To prepare mouse striatal primary microglial cultures, brain was removed from C57BL/6 mice of 2–4 days of age. Microglial cells were isolated following protocols described elsewhere [40, 49, 56] and grown in DMEM medium supplemented with 2 mM l-glutamine, 100 U/mL penicillin/streptomycin, MEM non-essential amino acids preparation (1/100), and 5% (v/v) heat-inactivated FBS (Invitrogen, Paisley, Scotland, UK). Briefly, striatum tissue was dissected, carefully stripped of its meninges, and digested with 0.25% trypsin for 20 min at 37 °C. The action of the proteolytic enzyme was stopped by adding an equal volume of culture medium (Dulbecco’s modified Eagle medium-F-12 nutrient mixture, fetal bovine serum 10%, penicillin 100 U/mL, streptomycin 100 μg/mL, and amphotericin B 0.5 μg/mL) with 160 μg/mL deoxyribonuclease I (all reagents from Invitrogen). Cells were brought to a single cell suspension by repeated pipetting followed by passage through a 100 μm-pore mesh and pelleted (7 min, 200×*g*). Glial cells were resuspended in medium and seeded at a density of 3.5 × 10^5^ cells/mL in 6-well plates for cyclic adenylic acid (cAMP) assays, in 12-well plates with coverslips for in situ proximity ligation assays (PLA), and in 96-well plates for mitogen-activated protein kinase (MAPK) activation experiments. Cultures were maintained at 37 °C in humidified 5% CO_2_ atmosphere and medium was replaced at DIV 2 and then once a week. At DIV 19-21 cells were treated with diluted trypsin [56] to obtain > 98% pure microglial cultures.

For neuronal primary cultures, the striatum from mouse embryos (E19) was removed and the neurons were isolated as described by [25] and plated at a density of circa 120,000 cells/cm^2^. The cells were grown in a neurobasal medium supplemented with 2 mM l-glutamine, 100 U/mL penicillin/streptomycin, and 2% (v/v) B27 supplement (Gibco) in a 6-, 12-, or 96-well plate for 19–21 days. Cultures were maintained at 37 °C in humidified 5% CO_2_ atmosphere and medium was replaced every 4–5 days.

Immunodetection of specific markers (NeuN for neurons and CD-11b for microglia) showed that neuronal preparations contained > 98% neurons and microglia preparations contained, at least, 98% microglial cells [39].

### Parkinson’s disease model generation, levodopa treatment, and dyskinesia assessment

All experiments were carried out in accordance with EU directives (2010/63/EU and 86/609/CEE) and were approved by the Ethical committee of the University of Santiago de Compostela. Similar to the approach elsewhere described [13], an experimental design using male Wistar rats was aimed at obtaining four group of animals as described below. Animals were 8-week-old at the beginning of the experimental procedure.

Details of model generation, protocol of drug administration, and behavioral analysis, performed by a blinded investigator, are given elsewhere [38, 47]. Surgery was performed on rats anesthetized with ketamine/xylazine (1% ketamine—75 mg/kg, and 2% xylazine—10 mg/kg). Lesions were produced in the right medial forebrain bundle to achieve complete lesion of the nigrostriatal pathway. The rats were injected with 12 μg of 6-OH-DA (to provide 8 μg of 6-hydroxy-DA free base; Sigma-Aldrich) in 4 μL of sterile saline containing 0.2% ascorbic acid. These were considered “lesioned” animals. Injection of vehicle lead to generation of naïve (or non-lesioned) animals.

The 6-OH-dopamine hemilesioned rat is considered a PD model. Amphetamine-induced rotation was tested in a bank of eight automated rotometer bowls (Rota-count 8, Columbus Instruments, Columbus, OH, USA) by monitoring full (360°) body turns in either direction. Right and left full body turns were recorded over 90 min following an injection of d-amphetamine (2.5 mg/kg i.p.) dissolved in saline. Rats that displayed more than six full body turns/min ipsilateral to the lesion were included in the study (this rate would correspond to more than 90% depletion of dopamine fibers in the striatum) [65].

Spontaneous use of forelimb can be measured by the cylinder test [28, 57]. Rats were placed individually in a glass cylinder (20 cm in diameter), and the number of left or right forepaw contacts were scored by an observer blinded to the animals’ identity and presented as left (impaired) touches in percentage of total touches. A control animal would thus receive an unbiased score of 50%, whereas lesion usually reduces performance of the impaired paw to less than 20% of total wall contacts.

Of the lesioned animals displaying parkinsonism-like behavior according to the above described tests (18 in total), 12 were chronically treated with l-DOPA daily for 3 weeks. A mixture of L-DOPA methyl ester (6 mg/kg) plus benserazide (10 mg/kg) was subcutaneously administered. The treatment reliably induces dyskinetic movements in some rats. As described in a previous report [13], abnormal involuntary movements (AIMs) were evaluated according to the rat dyskinesia scale described in detail elsewhere [5, 12, 33, 36, 47]. The severity of each AIM subtype (limb, orolingual, and axial) was assessed using scores from 0 to 4 (1, occasional, i.e., present < 50% of the time; 2, frequent, i.e., present > 50% of the time; 3, continuous, but interrupted by strong sensory stimuli; 4, continuous, not interrupted by strong sensory stimuli). Rats were classified as “dyskinetic” if they displayed a ≥ 2 score per monitoring period on at least two AIM subtypes. Animals classified as “non-dyskinetic” exhibited either no l-DOPA-induced abnormal involuntary movements or very mild/occasional ones [41]. Animals with low scores, i.e., either non-dyskinetic or dyskinetic, were discarded. In summary, four groups of animals were obtained: (i) non-lesioned, (ii) lesioned treated with vehicle, (iii) lesioned l-DOPA-treated becoming dyskinetic, and (iv) lesioned that upon l-DOPA treatment did not become dyskinetic. In every animal, tyrosine hydroxylase immunostaining was performed in sections taken post-mortem [19, 38]; selected animals undergoing 6-OH-dopamine treatment showed in the lesioned hemisphere a > 95% nigral dopaminergic denervation. Overall, 4 animals, those with better scores, were selected in each of following 4 groups: naïve, lesioned, lesioned/l-DOPA dyskinetic, and lesioned/l-DOPA non dyskinetic. PLA analysis (see below) was performed in different fields of striatal sections from each of the 16 finally selected animals. The striatum was delimited in sections using a bright field and images were captured within delimitation coordinates.

### Fusion proteins

Human cDNAs for AT_1_, AT_2_, and σ_1_ receptors cloned into pcDNA3.1 were amplified without their stop codons using sense and antisense primers harboring either BamHI and HindIII restriction sites to amplify AT_1_R and AT_2_R or BamHI and EcoRI restriction sites to amplify σ_1_ receptor. Amplified fragments were then subcloned to be in frame with an enhanced yellow fluorescent protein (pEYFP-N1; Clontech, Heidelberg, Germany) or a Rluc (pRluc-N1; PerkinElmer, Wellesley, MA) on the C-terminal end of the receptor to produce AT_1_R-YFP, AT_2_R-RLuc, AT_2_R-YFP, and σ_1_R-RLuc fusion proteins.

### Cell transfection

HEK-293T cells were transiently transfected with the corresponding cDNA by the polyethylenimine (PEI, Sigma-Aldrich, St. Louis, MO) method [18, 23]. Briefly, the corresponding cDNA diluted in 150 mM NaCl was mixed with PEI (5.5 mM in nitrogen residues) also prepared in 150 mM NaCl for 10 min. The cDNA-PEI complexes were transferred to HEK-293T cells and were incubated for 4 h in a serum-starved medium. Then, the medium was replaced by fresh supplemented culture medium and cells were maintained at 37 °C in a humid atmosphere of 5% CO_2_. Forty-eight hours after transfection, cells were washed, detached, and resuspended in the assay buffer.

### Immunocytochemistry

HEK-293T cells were seeded on glass coverslips in 12-well plates. On DIV 2, cells were transfected with AT_1_R-YFP cDNA (1 μg), AT_2_R-Rluc cDNA (1 μg), or both. On DIV 4, cells were fixed in 4% paraformaldehyde for 15 min and washed twice with PBS containing 20 mM glycine before permeabilization with PBS-glycine containing 0.2% Triton X-100 (5-min incubation). Cells were blocked during 1 h with PBS containing 1% bovine serum albumin. HEK-293T cells were labeled with a mouse anti-Rluc antibody (1/100; Millipore, Darmstadt, Germany) and subsequently treated with Cy3 conjugated anti-mouse (1/200; Jackson ImmunoResearch (red)) IgG (1 h each). The AT_1_R-YFP expression was detected by YFP’s own fluorescence. Controls using untransfected cells and cells incubated without the primary antibody are shown in Supplementary Figure S1. Samples were washed several times and mounted with 30% Mowiol (Calbiochem). Images were obtained in a Leica SP2 confocal microscope (Leica Microsystems) with the × 63 oil objective.

### Bioluminescence resonance energy transfer assays

For bioluminescence resonance energy transfer (BRET) assays, HEK-293T cells were transiently cotransfected with a constant amount of cDNA encoding for AT_2_R-RLuc (0.9 μg) and with increasing amounts of cDNA corresponding to AT_1_R-YFP (0.5 to 4 μg). For negative control, HEK-293T cells were transiently cotransfected with a constant amount of cDNA encoding for σ_1_-RLuc (0.75 μg) and with increasing amounts of cDNA corresponding to AT_2_R-YFP (0.1 to 4 μg). To control the cell number, sample protein concentration was determined using a Bradford assay kit (Bio-Rad, Munich, Germany) using bovine serum albumin (BSA) dilutions to prepare the standard absorbance versus concentration relationship. To quantify fluorescent proteins, cells (20 μg protein) were distributed in 96-well microplates (black plates with a transparent bottom) and fluorescence was read in a Fluostar Optima Fluorimeter (BMG Labtech, Offenburg, Germany) equipped with a high-energy xenon flash lamp, using a 10 nm bandwidth excitation filter at 485 nm. For BRET measurements, the equivalent of 20 μg of cell suspension was distributed in 96-well white microplates with white bottom (Corning 3600, Corning, NY) and 5 μM coelenterazine H (Molecular Probes, Eugene, OR) was added. One minute after adding coelenterazine H, BRET was determined using a Mithras LB 940 reader (Berthold Technologies, DLReady, Germany), which allows the integration of the signals detected in the short-wavelength filter at 485 nm and the long-wavelength filter at 530 nm. To quantify AT_2_R-RLuc expression, luminescence readings were performed 10 min after the addition of 5 μM coelenterazine H. MilliBRET units (mBU) are defined as:

$$ \mathrm{mBU}=\left[\ \frac{\uplambda_{530}\left(\mathrm{long}-\mathrm{wavelength}\ \mathrm{emission}\right)}{\uplambda_{485}\left(\mathrm{short}-\mathrm{wavelength}\ \mathrm{emission}\right)} - {C}_{\mathrm{f}}\ \right]\ \mathrm{x}\ 1000 $$

where *C*_f_ corresponds to [(long-wavelength emission)/(short-wavelength emission)] for the RLuc construct expressed alone in the same experiment.

### Detection of cytoplasmic calcium ion

HEK-293T cells were cotransfected with the cDNA for the Ang receptors AT_1_ (1 μg) and/or AT_2_ (1 μg) and the GCaMP6 calcium sensor (1 μg) [11] by the use of PEI method (Section “Cell Transfection”). Forty-eight hours post-transfection, HEK-293T cells plated in 6-well black, clear bottom plates, were incubated with Mg^2+^-free Locke’s buffer (154 mM NaCl, 5.6 mM KCl, 3.6 mM NaHCO_3_, 2.3 mM CaCl_2_, 5.6 mM glucose, 5 mM HEPES, pH 7.4) supplemented with 10 μM glycine. Receptor antagonists were added only 15 min before readings to allow efficient binding to receptors while avoiding unspecific or long-term noxious events. Receptor agonists were added right before readings as calcium level increase when mediated by Gq-coupled receptors is very quick. Fluorescence emission intensity of GCaMP6 was recorded at 515 nm upon excitation at 488 nm on the EnSpire® Multimode Plate Reader for 150 s every 5 s at 100 flashes per well.

### cAMP level determination

The analysis of cAMP levels was performed in HEK-293T cells transfected with cDNA for AT_1_ (1 μg) and/or AT_2_ (1 μg) receptors in primary cultures of striatal neurons or glia using the Lance Ultra cAMP kit (PerkinElmer). The optimal cell density to obtain an appropriate fluorescent signal was first established by measuring the TR-FRET signal as a function of forskolin concentration using different cell densities. Forskolin dose-response curves were related to the cAMP standard curve in order to establish which cell density provides a response that covers most of the dynamic range of cAMP standard curve. Two hours before the experiment, the medium was substituted by serum-starved DMEM medium. Cells (2000 HEK-293T cells, 4000 striatal neurons, or glial cells by well in 384-well microplates) growing in medium containing 50 μM zardaverine were pre-treated with the AT_1_R or AT_2_R antagonists (Candesartan and PD123319 respectively) or the corresponding vehicle at 24 °C for 15 min, and stimulated with the AT_1_R and/or AT_2_R agonists (Ang II and CGP-42112A respectively) for 15 min before adding 0.5 μM forskolin or vehicle, and incubating for an additional 15-min period. After 1 h, fluorescence at 665 nm was analyzed on a PHERAstar Flagship microplate reader equipped with an HTRF optical module (BMG Labtech). A standard curve for cAMP was obtained in each experiment.

### Extracellular signal-regulated kinases 1/2 phosphorylation

To determine extracellular signal-regulated kinases 1/2 (ERK1/2) phosphorylation, 40,000 HEK-293T cells transfected with cDNA for AT_1_R (1 μg) and/or AT_2_R (1 μg) or 50,000 striatal neurons or glial cells primary cultures were plated in each well of transparent Deltalab 96-well microplates. Two hours before the experiment, the medium was substituted by serum-starved DMEM medium. Then, cells were treated or not for 15 min with the selective antagonists Candesartan or PD123319 in serum starved DMEM medium followed by 7-min treatment with the selective agonists Ang II and/or CGP-42112A. Cells were then washed twice with cold PBS before the addition of lysis buffer (15-min treatment). Ten microliters of each supernatant were placed in white ProxiPlate 384-well microplates and ERK1/2 phosphorylation was determined using AlphaScreen®SureFire® kit (Perkin Elmer) following the instructions of the supplier and using an EnSpire® Multimode Plate Reader (PerkinElmer, Waltham, MA, USA).

### Dynamic mass-redistribution label-free assays

Cell signaling was explored using an EnSpire® Multimode Plate Reader (PerkinElmer, Waltham, MA, USA) by a label-free technology. Cellular cytoskeleton redistribution movement induced upon receptor activation were detected by illuminating the underside of the plate with polychromatic light and measured as changes in wavelength of the reflected monochromatic light that is a sensitive function of the index of refraction. The magnitude of this wavelength shift (in picometers) is directly proportional to the amount of dynamic mass redistribution (DMR). To determine the label free-DMR signal, 10,000 HEK-293T cells transfected with cDNA for AT_1_R (1 μg) and/or AT_2_R (1 μg) receptors or 10,000 striatal neurons or glial cells primary cultures were plated on each well of transparent 384-well fibronectin-coated microplates to obtain 70–80% confluent monolayers, and kept in the incubator for 24 h. Previous to the assay, cells were washed twice with assay buffer (HBSS with 20 mM HEPES, pH 7.15, 0.1% DMSO) and incubated in the reader for 2 h in 30 μL/well of assay buffer at 24 °C. Hereafter, the sensor plate was scanned and a baseline optical signature was recorded for 10 min before adding 10 μL of antagonists (Candesartan or PD123319) dissolved in assay buffer, followed by the addition, 30 min later of 10 μL of selective agonists (Ang II and/or CGP-42112A) also dissolved in assay buffer. DMR responses induced by the agonist were monitored for a minimum of 3600 s.

### Proximity ligation assay

Detection in natural sources of clusters formed by AT_1_ and AT_2_ receptors was addressed in primary cultures of glial cells and rat brain sections.

Rats were processed for histological analysis as it follows. Animals were injected an overdose of chloral hydrate and transcardial perfusion fixation quickly undertaken using cold 4% paraformaldehyde in 0.1 M phosphate buffer (PB), pH 7.4. Brains were removed, washed, and cryoprotected in the same buffer containing 20% sucrose. Serial 40-μm-thick coronal sections were then cut with a freezing microtome and those containing the striatum were collected in cryoprotectant solution.

Cells were grown on glass coverslips, fixed in 4% paraformaldehyde for 15 min, washed with PBS containing 20 mM glycine to quench the aldehyde groups, permeabilized with the same buffer containing 0.05% Triton X-100 for 5 to 15 min, and washed with PBS.

Cells and sections were then similarly processed. After 1-h incubation at 37 °C with the blocking solution in a pre-heated humidity chamber, samples were incubated overnight at 4 °C with a mixture of a mouse monoclonal anti-AT_1_R antibody (1/100, sc-515884, Santa Cruz Biotechnology, Texas, USA), a rabbit monoclonal anti-AT_2_R antibody (1/100, ab92445, Abcam, Cambridge, UK), and Hoechst (1/100 from stock 1 mg/mL; Sigma-Aldrich) to stain the nuclei. The antibodies were validated following the method in the technical brochure of the vendor with fairly similar results and also by immunofluorescence in HEK-293T cells (transfected versus untransfected). Samples from KO animals were not available for validation.

Cells or brain sections were further processed using the proximity ligation assays (PLA) probes detecting primary antibodies (Duolink In Situ PLA probe Anti-Mouse plus and Duolink In Situ PLA probe Anti-Rabbit minus) (1/5 v:v for 1-hour at 37 °C). Ligation and amplification were done as indicated by the supplier (Sigma-Aldrich) and cells were mounted using the mounting medium 30% Mowiol (Calbiochem). To detect red dots corresponding to AT_1/2_Hets, samples were observed in a Leica SP2 confocal microscope (Leica Microsystems, Mannheim, Germany) equipped with an apochromatic × 63 oil-immersion objective (N.A. 1.4), and a 405 nm and 561 nm laser lines. For each field of view, a stack of two channels (one per staining) and 3 Z-planes with a step size of 1 μm were acquired. The Andy’s Algorithm [32], a specific ImageJ macro for reproducible and high-throughput quantification of the total PLA foci dots and total nuclei, was used for data analysis.

### Statistical analysis

The data in graphs are the mean ± SEM. GraphPad Prism software version 7 (San Diego, CA, USA) was used for data fitting and statistical analysis. The test of Kolmogorov-Smirnov with the correction of Lilliefors was used to evaluate normal distribution and the test of Levene to evaluate the homogeneity of variance. The Student’s t test and one-way ANOVA were used for comparing, respectively, two or > 2 means. When indicated, Bonferroni’s method was used as a post-hoc test for multiple comparisons. Significant differences were considered when the *p* value was < 0.05.

## Results

### Functionality of AT_1/2_Hets in a heterologous expression system

**Fig. 1:**
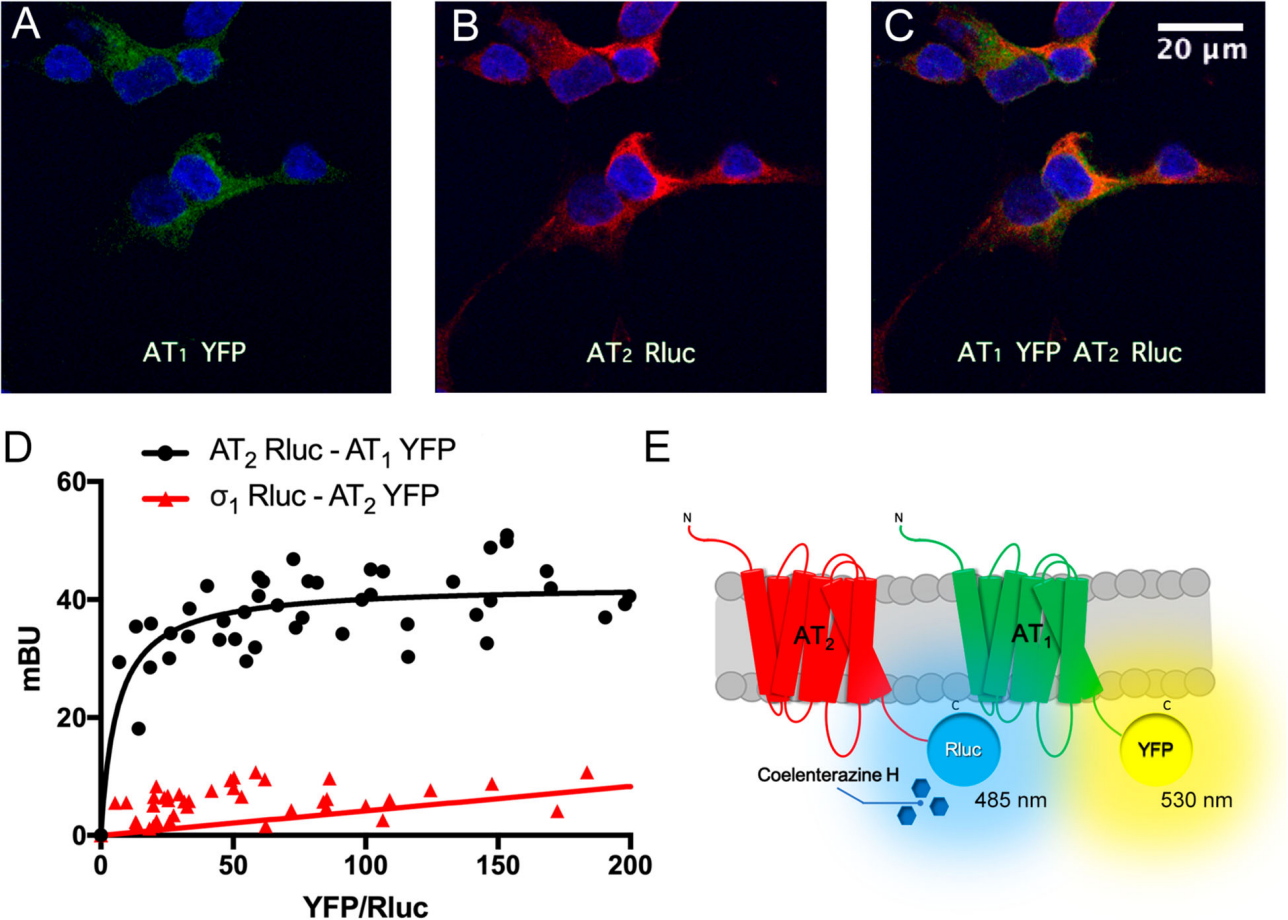
Human AT_1_ and AT_2_ receptors interact in a heterologous expression system. **a**–**c** Immunocytochemistry assays were performed in HEK-293T cells expressing AT_1_R-YFP (1 μg cDNA), which was detected by its own yellow fluorescence (green), and AT_2_R-Rluc (1 μg cDNA), which was detected by a mouse anti-Rluc antibody and a secondary Cy3 anti-mouse antibody (red). Colocalization is shown in yellow. Cell nuclei were stained with Hoechst (blue). Scale bar: 20 μm. **d** BRET assays were performed in HEK-293T cells transfected with a constant amount of cDNA for AT_2_R-Rluc (0.9 μg) or σ_1_R-Rluc (0.75 μg) (as negative control) and increasing amounts of cDNA for AT_1_R-YFP (0.5 to 4 μg) or AT_2_R-YFP (0.1 to 4 μg) (as negative control). Values are the mean ± S.E.M. of 8 independent experiments performed in duplicates. **e** Schematic representation of BRET assay: the occurrence of energy transfer depends on the distance between the BRET donor (Rluc) and the BRET acceptor (YFP)

Interactions between AT_1_ and AT_2_ receptors have been previously reported [14, 48]. Hence, we first investigated whether in HEK-293T cells and in our assay conditions AT_1_ and AT_2_ receptors may form heteromers. We analyzed the colocalization of angiotensin receptors at the plasma membrane by using HEK-293T cells coexpressing AT_1_R and AT_2_R fused to, respectively, the yellow fluorescent protein (YFP) and Renilla luciferase (Rluc). The proper traffic of fusion proteins to the cell membrane was confirmed by immunocytochemical analysis (Fig. 1a, b). The high degree of colocalization between AT_1_R-YFP and AT_2_R-Rluc in the plasma membrane and in the cytosol is shown in yellow (Fig. 1c). To know whether a direct interaction between AT_1_ and AT_2_ receptors is possible, BRET assays were performed in HEK-293T cells expressing a constant amount of a fusion protein consisting of AT_2_R and Renilla Luciferase (AT_2_R-Rluc) and increasing amounts of AT_1_R fused to YFP (AT_1_R-YFP). The saturation curve in Fig. 1d indicates close proximity between the two Ang receptors. The BRET_max_ and BRET_50_ values were 42 ± 1 mBU and 6 ± 2, respectively. When HEK-293T cells were transfected with a constant amount of cDNA for σ1-Rluc and increasing amounts of cDNA for AT_1_R-YFP, a linear response was observed indicating a nonspecific interaction of this negative control (Fig. 1d). These results confirm that the two Ang receptors may form heteromers in living HEK-293T cells. The proper functionality of fusion proteins was confirmed by cAMP assays (data not shown). A schematic representation of the technique is shown in Fig. 1e.

**Fig. 2:**
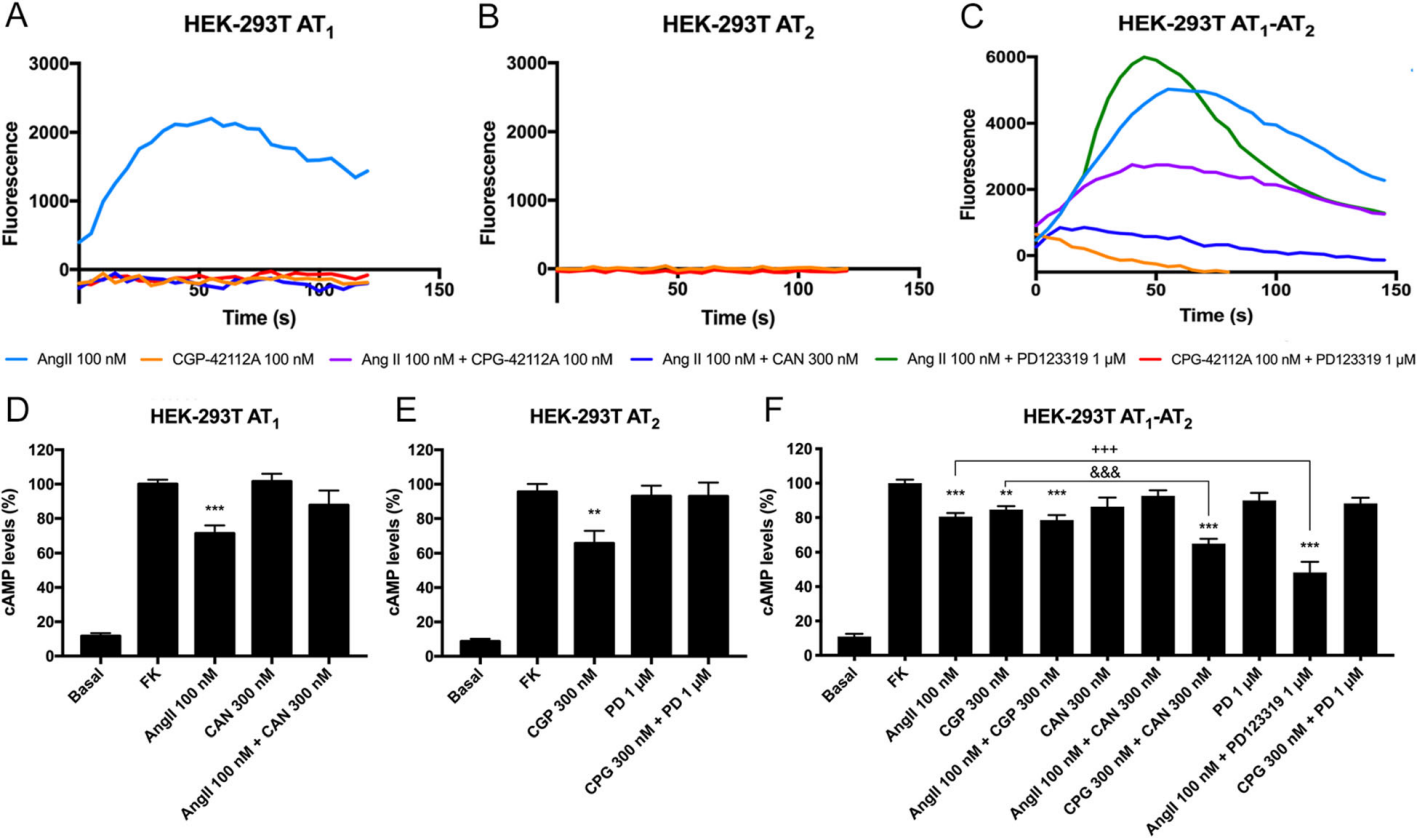
Functional characterization in HEK-293T cells expressing the AT_1_R-AT_2_R heteromer. HEK-293T cells were pretreated with selective receptor antagonists (300 nM candesartan for AT_1_R or 1 μM PD123319 for AT_2_R) and subsequently treated with selective agonists (100 nM angiotensin II for AT_1_R and 300 nM CGP-42112A for AT_2_R) **a–c** Cytosolic calcium detection assay were performed in HEK-293T cells transfected with the cDNAs for an engineered calcium sensor, 6GCaMP (1 μg), AT_1_R (1 μg), and/or AT_2_R (1 μg). Values are the mean ± S.E.M. of 5 independent experiments performed in duplicates. **d–f** Intracellular cAMP levels were determined by TR-FRET as described in Methods. HEK-293T cells were transfected with cDNAs for AT_1_R (1 μg) and/or AT_2_R (1 μg). When G_i_ coupling was assessed, decreases in [cAMP] were determined using 0.5 μM forskolin added 15 min after the agonists stimulation. Values are the mean ± S.E.M. of 6 independent experiments performed in triplicates. In cAMP one-way ANOVA followed by Bonferroni’s multiple comparison post-hoc test were used for statistical analysis. Signaling output was the dependent variable and the different treatments were the independent variables. (*p < 0.05, **p < 0.01, ***p < 0.001 versus forskolin treatment; +++*p* < 0.001 versus Ang II treatment; &&&*p* < 0.001 versus CGP-42112A treatment)

To characterize the AT_1/2_Het functionality, signaling assays were performed in single-transfected HEK-293T cells and in cotransfected AT_1/2_Hets-expressing cells. Cytosolic calcium levels, cAMP determination, ERK1/2 phosphorylation, and label-free DMR assays were performed after treatment with AT_1_R and/or AT_2_R ligands. Consistent with Gq coupling of AT_1_R, treatment of AT_1_R-expressing cells with Ang II led to a marked increase in cytosolic calcium levels. The effect was receptor-mediated, as it was blocked by candesartan, the AT_1_R antagonist (Fig. 2a). In AT_2_R expressing cells, the AT_2_R agonist CGP-42112A did not induce mobilization of the ion. These results agree with AT_2_R not engaging Gq proteins. In cotransfected cells, CGP-42112A did not produce any effect but reduced the response peak produced by Ang II. Hence, within the AT_1/2_Het, AT_2_R stimulation inhibits the AT_1_ receptor signaling. Unlike the selective AT_1_R antagonist, candesartan, which blunted the agonist effect, the selective AT_2_R antagonist, PD123319, potentiated the AT_1_R-mediated effect (Fig. 2c).

Consistent with AT_2_R coupling to Gi, CGP-42112A reduced the forskolin-induced cAMP cytosolic levels in AT_2_R single transfected cells. Interestingly, in AT_1_R-expressing cells, Ang II treatment also reduced the forskolin-induced cAMP levels. These effects can be explained by AT_1_R coupling not only to Gq, but also to Gi proteins. These effects were receptor-mediated and specific as they were blocked by the corresponding antagonist (Fig. 2d, e). Analysis of cAMP-PKA signaling in cotransfected cells is more complex. On the one hand, all agonists reduced forskolin-induced [cAMP] and each antagonist blocked the effect of the corresponding agonist. Simultaneous administration of Ang II and CGP-42112A did not result in an additive effect. On the other hand, the antagonist of the AT_2_R enhanced the effect induced by the AT_1_R agonist and vice-versa, the antagonist of the AT_1_R enhanced the effect of the AT_2_R agonist (Fig. 2e). These latter results fit with data from calcium release experiments assays where the antagonist of the AT_2_R potentiated the action of Ang II. Then, it seems that both angiotensin receptors regulate one another via heteromerization. The negative regulation can be reversed and the antagonist of one receptor can even reinforce the output due to activation of the partner receptor.

**Fig. 3:**
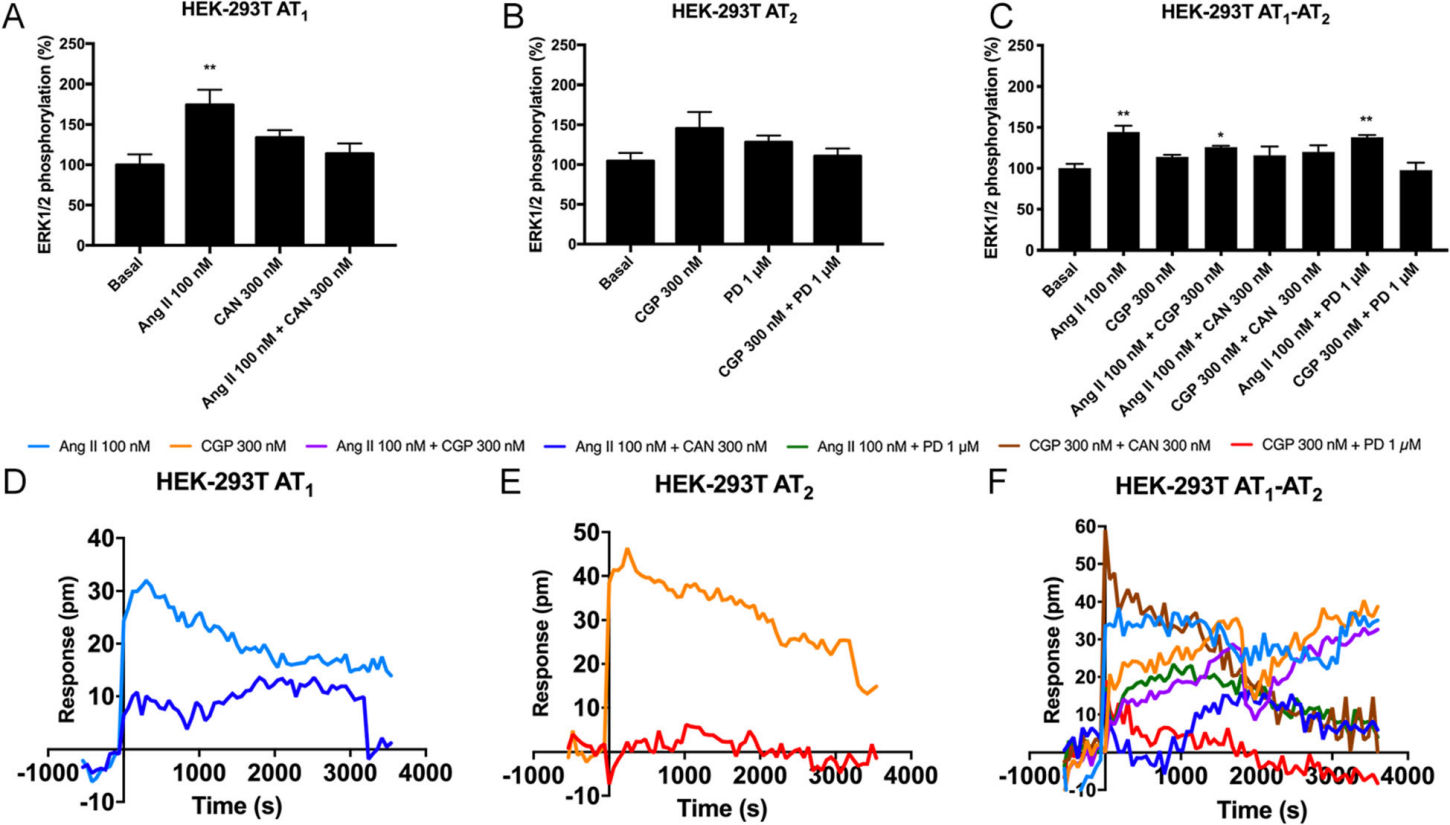
Functional characterization of AT_1_R-AT_2_R heteromer in HEK-293T cells. HEK-293T cells were transfected with cDNAs for AT_1_R (1 μg) and/or AT_2_R (1 μg). Cells were pretreated (15 min) with selective receptor antagonists (300 nM candesartan for AT_1_R or 1 μM PD123319 for AT_2_R receptors) and subsequently treated with selective agonists (100 nM angiotensin II for AT_1_R and 300 nM CGP-42112A for AT_2_R receptors). **a–c** ERK1/2 phosphorylation was analyzed using an AlphaScreen®SureFire® kit (Perkin Elmer). Values are the mean ± S.E.M. of 5 independent experiments performed in duplicates. One-way ANOVA followed by Bonferroni’s multiple comparison post-hoc test were used for statistical analysis. Signaling output was the dependent variable and the different treatments were the independent variables (*p < 0.05, **p < 0.01, ***p < 0.001; versus vehicle treatment (basal)). **d–f** DMR tracings represent the picometer-shifts of reflected light wavelength over time. Values are the mean ± S.E.M. 8 independent experiments performed in triplicates

In AT_1_R-expressing cells, a marked increase in agonist-induced ERK1/2 phosphorylation that was blocked by candesartan was observed, while in AT_2_R-expressing cells, a mild non-significant effect was obtained by treatment with the agonist, CGP-42112A (Fig. 3a, b). Remarkably, AT_1/2_Hets expression in cotransfected cells led to a significant increase in ERK1/2 phosphorylation upon Ang II treatment. The selective AT_2_R agonist was still unable to produce any significant effect but, in combined treatments, it markedly reduced the effect induced by Ang II (Fig. 3c). Therefore, these results are in the same line with that observed in calcium release and cAMP accumulation, indicating that both receptors inhibit one another in the AT_1/2_Het.

DMR was modified by agonists in both types of single-transfected cells. In AT_1_R-expressing cells, the antagonist could not revert totally the effect of the agonist, indicating that part of the output signal is due to off-site action of Ang II (Fig. 3d). In contrast, the effect of CGP-42112A on AT_2_R-expressing was totally blocked by PD123319 (Fig. 3e). In cotransfected cells, the results indicate that both angiotensin receptors show a characteristic signal that is blocked by selective antagonists. However, coactivation in these cells produced an important decrease in the signal. This effect is named negative crosstalk and it is similar to that observed in MAPK phosphorylation assays (Fig. 3e). The enhancement by candesartan of the effect of CGP-42112A, the AT_2_R agonist, was also noticed.

In summary, these results indicate a particular functionality of the AT_1/2_Het, namely Ang is able to engage two different signaling pathways. This dual effect is regulated by activation of the partner receptor within the AT_1/2_Het as (i) activation of AT_1_R blocks AT_2_R agonist induced effect and vice-versa and (ii) AT_1_R antagonist releases the inhibition exerted by AT_2_R over AT_1_R and vice-versa.

## Functionality of AT_1_R and AT_2_R in primary cultures of striatal neurons

**Fig. 4:**
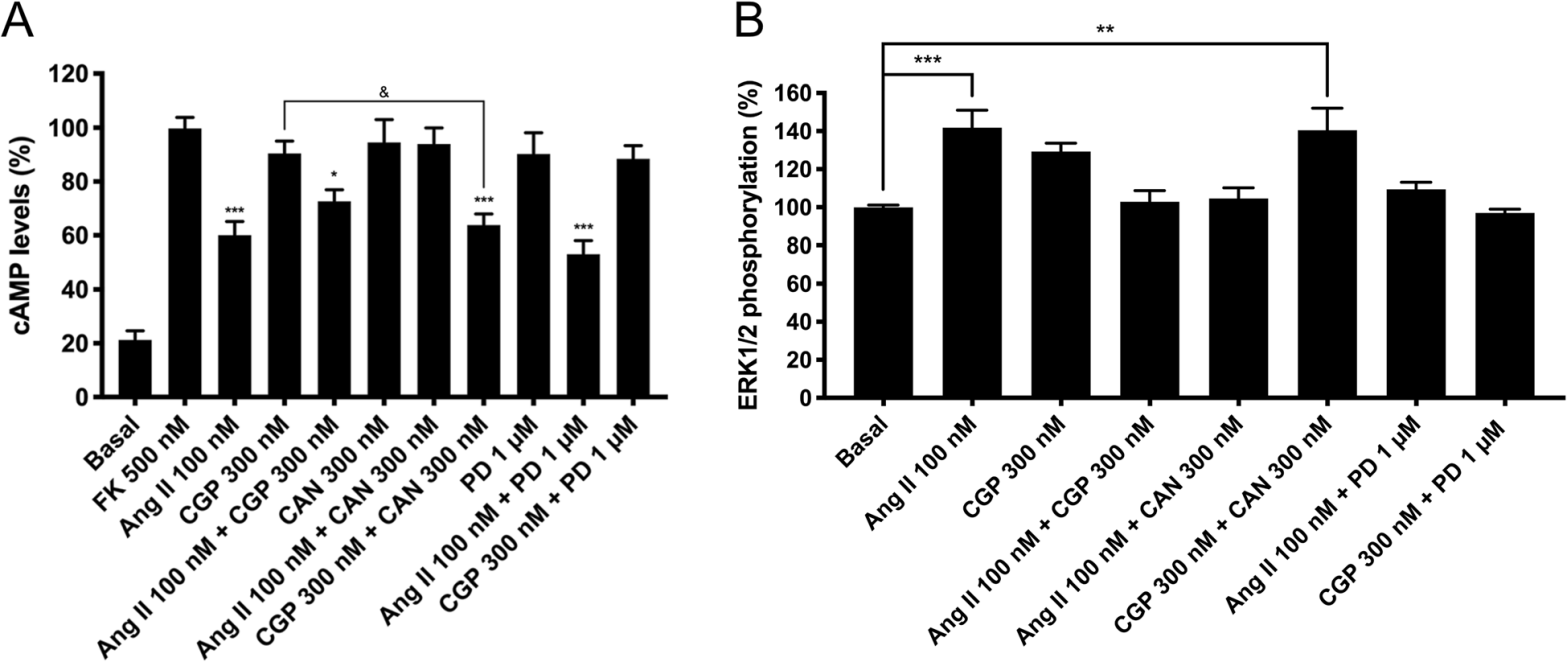
AT_1_R-AT_2_R heteromer functionality in primary cultures of striatal neurons. For cAMP (**a**) or ERK1/2 phosphorylation (**b**), cells were pretreated (15 min) with selective receptor antagonists (300 nM candesartan for AT_1_R or 1 μM PD123319 for AT_2_R) and subsequently treated with selective agonists (100 nM angiotensin II for AT_1_R and/or 300 nM CGP-42112A for AT_2_R). Values are the mean ± S.E.M. of 5 independent experiments performed in triplicates. One-way ANOVA followed by Bonferroni’s multiple comparison post-hoc test were used for statistical analysis. Signaling output was the dependent variable and the different treatments were the independent variables. (&*p* < 0.05 versus CGP-42112A treatment; *p < 0.05, **p < 0.01, ***p < 0.001 versus forskolin treatment in cAMP determinations or versus vehicle treatment (basal) in pERK determinations)

On the basis of the above-described relevance of Ang receptors in motor control and as potential targets to combat PD, we investigated Ang receptor-mediated signaling in primary cultures of neurons isolated from mouse striatum. Reduction of forskolin-induced [cAMP] was obtained using Ang II, while no sign of Gi-coupled AT_2_R was detectable (Fig. 4a). Interestingly, in simultaneous treatment with agonists, the lower decrease of forskolin-induced [cAMP] indicated the presence of AT_2_R whose activation reduced AT_2_R-mediated signaling. In the presence of the AT_1_R antagonist, candesartan, the AT_2_R agonist, CGP-42112A, induced a significant decrease in forskolin-induced cAMP levels (& in Fig. 4a). These results indicate that in the AT_1/2_Het, the AT_2_R signal is inhibited by AT_1_R expression and this effect is counteracted by AT_1_R antagonists. Similar results were confirmed in experiments of ERK1/2 phosphorylation determination in cultured striatal neurons, where cells responded (moderately) to Ang II and non-significantly to CGP-42112A (Fig. 4b). However, candesartan pretreatment potentiated AT_2_R signal. In agreement with the results in HEK-293T cells, cotreatment with agonists induced a lower signal to that induced by angiotensin II, indicating a negative crosstalk effect in both cAMP and MAPK phosphorylation signaling pathways. Overall, these cells express AT_1/2_Hets with similar functional characteristics to that observed in cotransfected HEK-293T cells.

### Functionality of AT_1_R and AT_2_R in primary cultures of striatal microglia: expression of AT_1/2_Hets

**Fig. 5:**
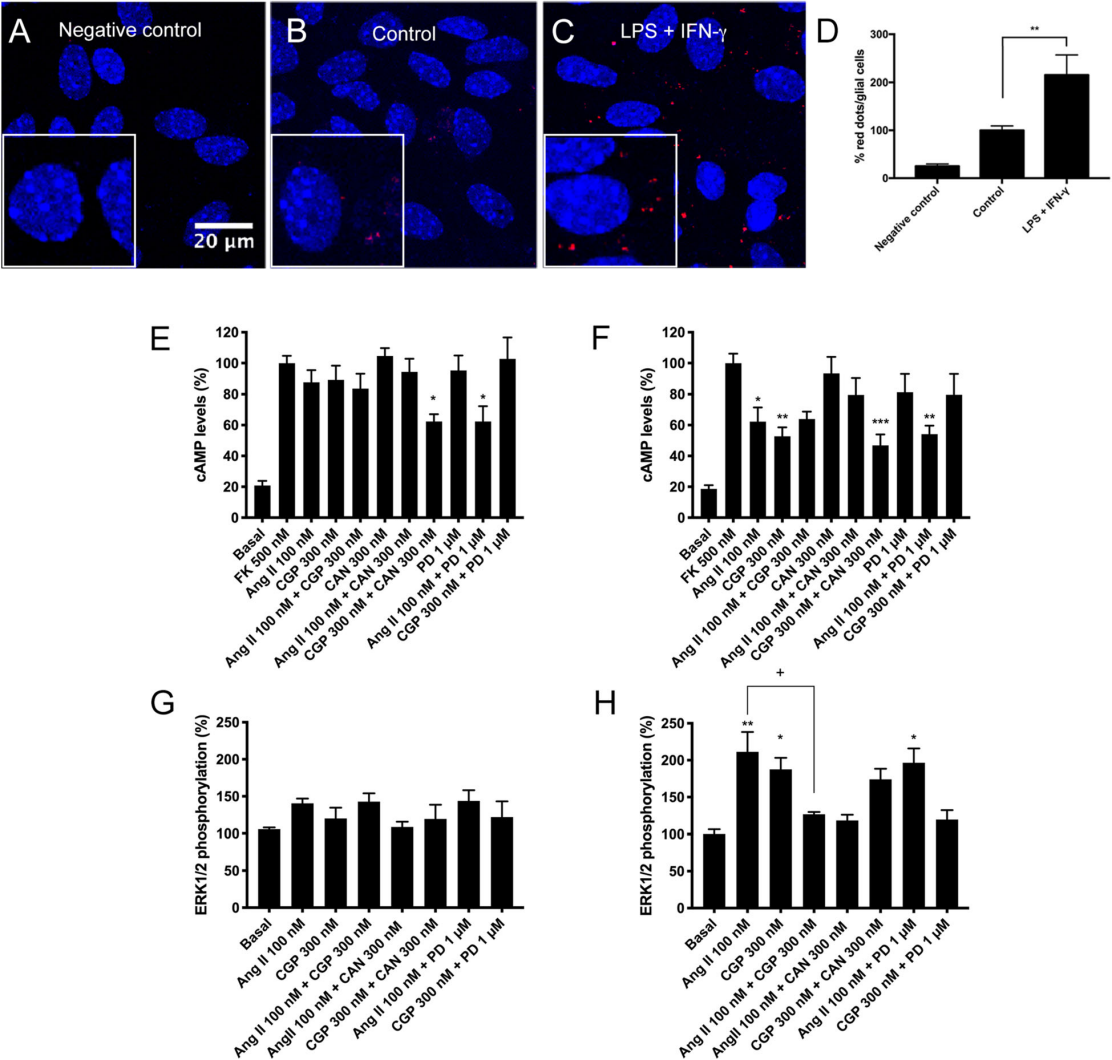
AT_1_R-AT_2_R heteromer functionality in microglial primary cultures treated with LPS and IFN-γ. **a**–**c** Expression of AT_1_R/AT_2_R heteromers in primary microglial cultures were determined by PLA, which was performed using specific primary antibodies against AT_1_ and AT_2_ receptors (confocal microscopy images (stacks of 3 consecutive planes) show heteroreceptor complexes as red clusters and Hoechst-stained nuclei (blue)). Scale bar: 20 μm. **d** Bar graph showing the percentage of red dots/cell respect non-treated cells; mean ± S.E.M of counts in 5–7 different fields (*n* = 5; **p < 0.01; Student’s *t* test versus the control condition). **e, f** Microglial cultures were incubated for 48 h in the absence (left) or in the presence (right) of 1 μM LPS and 200 U/mL IFN-γ. Microglial cells were pretreated (15 min) with selective receptor antagonists (300 nM candesartan for AT_1_R or 1 μM PD123319 for AT_2_R receptors) and subsequently with the specific agonists (100 nM angiotensin II for AT_1_R and 300 nM CGP-42112A for AT_2_R receptors). cAMP (**e**-**f**) and ERK1/2 phosphorylation (**g-h**) were subsequently measured. Values are the mean ± S.E.M. of 5 independent experiments performed in triplicates. One-way ANOVA followed by Bonferroni’s multiple comparison post-hoc test were used for statistical analysis. Signaling output was the dependent variable and the different treatments were the independent variables. (+*p* < 0.05 versus Ang II treatment in pERK determinations; and *p < 0.05, **p < 0.01, ***p < 0.001; versus forskolin treatment in cAMP measurements or versus vehicle treatment (basal) in pERK measurements)

Before assessing the functionality of Ang receptors in resting and LPS + IFN-γ activated microglia isolated from striatum, we used in situ PLA to assess the expression of AT_1/2_Hets; PLA is instrumental to detect clusters of interacting proteins in natural sources. Figure 5b shows that resting cells have a few number of red dots due to AT_1/2_Hets, while the negative control show a negligible number of red dots (Fig. 5a). Interestingly, the red label was markedly increased in activated cells, as shown in Fig. 5c and in the bar graph of Fig. 5d indicating that AT_1/2_Hets expression increases in activated microglia. We then performed cAMP level determination and ERK1/2 phosphorylation assays in resting and activated microglial cells. The functionality of AT_1_R, AT_2_R, and/or AT_1/2_Hets in resting cells was very low. But in cAMP assays, pretreatment with candesartan potentiated AT_2_R induced signaling, and PD123319 potentiated AT_1_R functionality (Fig. 5e, g). In contrast, the angiotensin-receptor-mediated signaling was more robust in LPS + IFN-γ activated cells (Fig. 5f, h). In cAMP assays, the agonist of the two receptors reduced the forskolin-induced levels of this second messenger, although receptor costimulation did not lead to any additive effect. As it occurred in HEK-293T cells and neuronal primary cultures, antagonist pretreatments potentiated the partner receptor signal. On the other hand, the agonist of any of the two receptors activated the MAPK signaling pathway, while simultaneous stimulation completely blunted the ERK1/2 phosphorylation effect in activated microglia. In this signaling pathway, antagonists blocked the cognate receptor and did not potentiate the activation of the partner receptor in the heteromer. Taken together, these results indicate that AT_1/2_Hets are significantly expressed in activated microglia showing the same properties than those displayed in heterologous system.

### Expression AT_1/2_Hets in the striatum of parkinsonian and dyskinetic rats

**Fig. 6:**
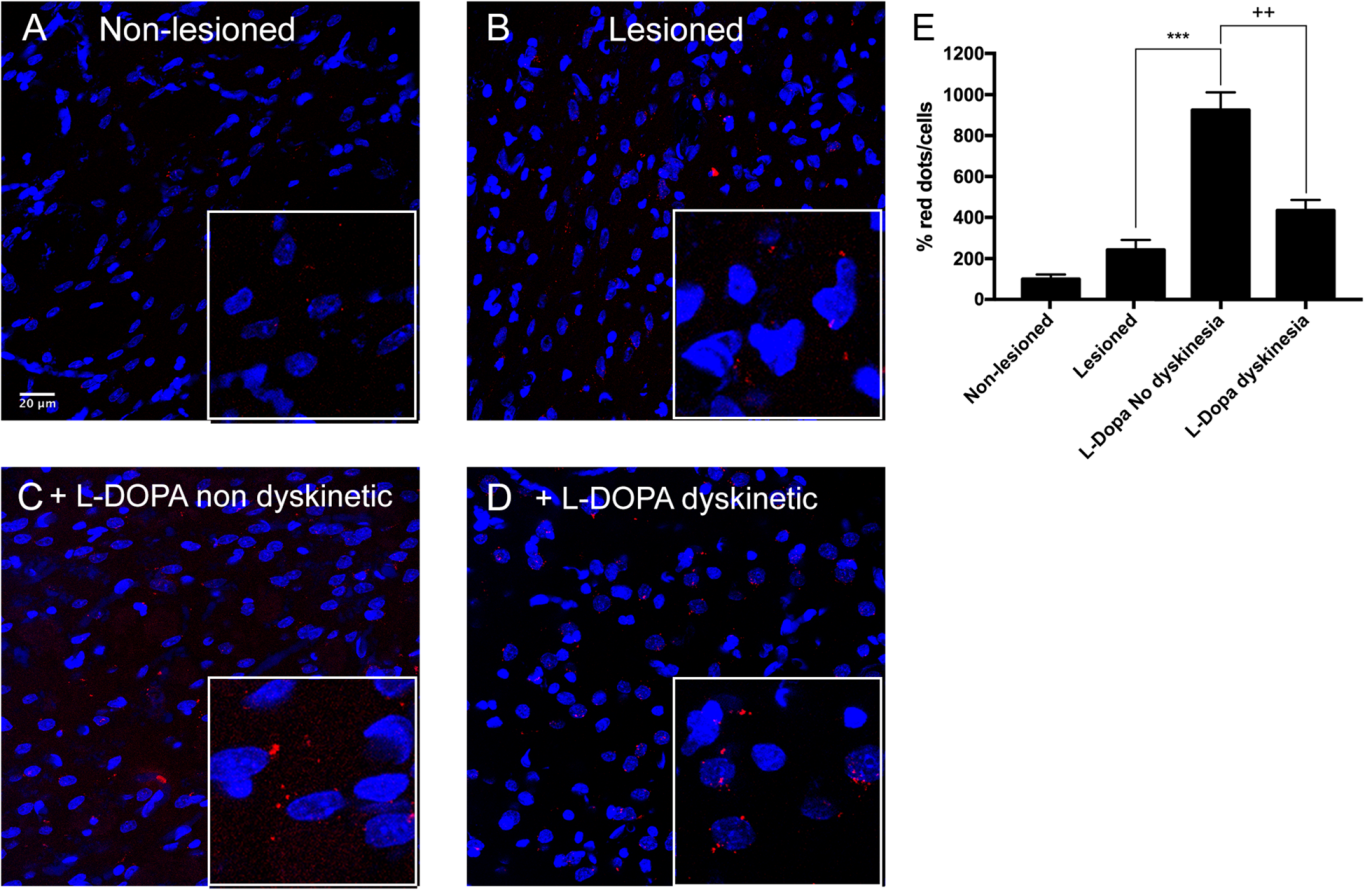
AT_1_R-AT_2_R heteromer expression in brain striatal sections of Parkinson’s disease (PD) rat model. **a–d** PLA assays in striatal sections from the 6-OH-dopamine PD rat model, non-lesioned (**a**), lesioned (**b**), and lesioned plus chronically treated with l-DOPA and either lacking (**c**) or displaying (**d**) dyskinesias. Confocal microscopy images (stacks of 3 consecutive planes) show heteroreceptor complexes as red clusters and Hoechst-stained nuclei (blue). Scale bar: 20 μm. **e** Bar graph showing the percentage of red dots/cell. Data are the mean S.E.M . of counts in 9–12 different fields per animal (*n* = 4 per group). One-way ANOVA followed by Bonferroni’s post-hoc multiple comparison tests were used to compare the red dots/cell values. The number of clusters (*r*) was the dependent variable and the four animal groups treatments were independent variables (***p < 0.001; versus lesioned condition, ++*p* < 0.01; versus l-DOPA non-dyskinesia condition)

Striatal sections of naïve and of 6-OH-dopamine hemilesioned rats, treated or not with l-DOPA and divided into l-DOPA/dyskinetic or resistant to l-DOPA-induced dyskinesia were prepared as described in methods. PLA assays were performed simultaneously and in identical conditions to detect the occurrence and the amount of AT_1/2_Hets. A representative image of each of the conditions is shown in Fig. 6a–d, while quantitation is shown in the form of bar graph in Fig. 6e. Whereas the amount of AT_1/2_Hets was negligible in the non-lesioned striatum, the lesioned one displayed more receptor clusters. l-DOPA/dyskinetic animals showed a two-fold increase if compared with the ipsilateral hemisphere of lesioned rats. Remarkably, animals resistant to l-DOPA-induced dyskinesia had a circa 10-fold increase in the amount of AT_1/2_Hets (compared to control, non-lesioned hemisphere). These results show that, in this specific PD model, of maximal dopaminergic denervation similar to that observed in advanced stages of the disease, there is striatal expression of the heteromer that is increased upon l-DOPA administration. Remarkably, a much higher increase in expression was found in l-DOPA-treated animals that did not become dyskinetic.

## Discussion

Details of peripheral RAS have been instrumental to develop a successful therapy to combat hypertension. The knowledge of RAS in the CNS is still fragmentary but with repercussions in dopaminergic neurotransmission and potential in PD and/or PD therapy-related dyskinesias. One of the added values of any future PD intervention based in central RAS is the safety of angiotensin receptor antagonists that have been used for decades in hypertensive patients. While no double-blind placebo controlled clinical trials have been designed, epidemiological studies addressing the risk of hypertensive patients taking angiotensin receptor antagonists have been performed. The fact that not all the drugs are able to cross the blood-brain barrier complicates data analysis. Anyhow, some of the reported results appear promising and using ß-blockers as a control and 65,001 hypertensive patients for > 4 years [34] showed that the use of certain antihypertensives (angiotensin receptor blockers included) associates with reduced PD risk. The still unsolved question is whether such drugs may be of benefit in improving symptoms, avoiding l-DOPA-induced dyskinesias and/or in delaying the progression of the disease.

First of all, it has been described that the global effects of angiotensin on AT_1_ and AT_2_ receptors are opposite. In several tissues, overactivity of the AT_1_ receptor has been linked to aging-related pro-inflammatory changes ([16]; Labandeira-Garcia et al., [29, 30]). Different mechanisms have been proposed to explain counteracting effects of AT_2_R on AT_1_R signaling (see [43, 44], and [59] for review). Although the issue is complex, the expression of AT_2_R in brain suggests a relevant role in the regulation of neuroinflammation. On the one hand, as earlier indicated, the two angiotensin receptors may interact leading to receptor heteromers with particular properties such as regulating SERCA activity [14, 48]. On the other hand, several heteromers formed by angiotensin receptors have been described. Among other examples [58], it has been reported that apelin and AT_1_ receptors interact and that the resulting heteromers mediate the apelin inhibition of AT_1_R-mediated actions. MAS protooncogene, the novel player in RAS research, rescues a defective AT_1_R, likely by forming heterodimers [55]. The discovery of interaction between AT_1_R and the most abundant receptor in the CNS, namely the cannabinoid CB_1_ receptor, has led to hypothesize that these functional units may lead to pathogenic actions when Ang II is produced. However, the potential toxicity was studied in hepatic cells in relation to ethanol consumption but it was not assayed in neuronal cell models [53]. The AT_1_R may form complexes with a variety of adrenergic receptors [4, 21], although their relevance to CNS physiology seems scarce. Less data are available for the AT_2_R receptor that may interact (in the periphery) with MAS and with bradykinin B2 receptors [1, 35, 44, 63]. In addition, AT_1/2_Hets may form dimers that may eventually interact with MAS or with bradykinin B2 receptors in cells in which three of these receptors are expressed together [10, 54]. One of the relevant results in this study is the demonstration in a rat model of microglial AT_1/2_Hets expression and its upregulation correlating with PD and with ulterior treatment with L-DOPA, the most extended therapeutic agent in PD [9, 24, 42].

What is relevant for PD pathophysiology and disease progression is to delay the death of the approximately 30% nigral dopaminergic neurons that are left at the time of PD diagnosis. Glia in general, and microglia in what concerns to neuroinflammation, are key to preserve neurons from death. AT_1_Rs have been proposed as targets to reduce chronic neuroinflammation [26, 27, 50] as that occurring in PD. The general idea is that activation of the AT_1_R is detrimental, for instance by a microglia-mediated enhancement of neuronal loss in status epilepticus induced in rats [60]. In a previous study performed in the SN of rats we showed that angiotensin-induced Rho-kinase activation was involved in NADPH-oxidase activation, which, in turn, was involved in angiotensin-induced Rho-kinase activation [51]. In addition, a prevention of astrocyte activation and promotion of hippocampal neurogenesis has been attributed to AT_1_R blockade and subsequent prevention of NFкB and MAP kinase signaling and activation of Wnt/β-catenin signaling [7]. In our experimental conditions performed with already activated striatal microglia, MAP kinase signaling is suppressed by combined treatment with AT_1_ and AT_2_ receptor agonists. In the retina, AT_1_R activation results in regulating microglial activation thus suggesting that Ang II may have important implications in diabetic retinopathy [46]*.* To our knowledge, the expression of AT_1/2_Hets in retinal cells has not been addressed yet. In agreement with the occurrence of a RAS opposite arm consisting of AT_2_R (also of Mas receptor) (Labandeira-Garcia et al., [29, 30]), AT_2_R activation attenuates microglial activation in an autoimmune encephalomyelitis rodent model [62]. Activation of the receptor is neuroprotective in a model of ischemia induced in conscious rats [37]. Similarly, the report by Bennion et al. [6] proves that AT_2_R activation in neurons and glial cells affords long-term neuroprotection in stroke, by both direct and indirect mechanisms. Recent studies show that the receptor prevents/attenuates pro-inflammatory microglial activity via protein phosphatase 2A-mediated inhibition of protein kinase C [8].

To our knowledge, the AT_1_-bradikinin B2 heteroreceptor complex was the first to be associated to a peripheral-affecting disease. In fact, the expression of the heteromer was increased thus mediating a higher Ang responsiveness in preeclampsia, a disease that markedly alters blood pressure in pregnancy [2]. The heteromer physiological function involves diverse signaling pathways and a variety of cells events such as phosphorylation of c-Jun terminal kinase and enhanced production of nitric oxide and a second messenger, cGMP [1]. An unbalanced proportion of AT_1_-bradikinin B2 receptor heteromers alters activation of cognate G-proteins and receptor desensitization [3, 64]. We have collected data on differential expression of dopamine-receptor-containing heteromers in PD and the conclusion is that very often the expression of heteromers is altered in one or different stages of the disease. Usually, the expression of those heteromers in dyskinesia is lower than in PD animals treated with l-DOPA but not displaying dyskinesias. Hence, this is the first example in which the already enhanced expression of heteromers in parkinsonian conditions is further increased in animals that were not rendered dyskinetic by l-DOPA treatment. These results are relevant as antagonists of angiotensin are considered as having potential to both improve PD symptoms and minimize l-DOPA-induced dyskinesias.

As in any other study focused on a neurodegenerative disease, there are a number of limitations. Among them we are extrapolating to “adult” cells using primary cells obtained from fetuses of neonates. Although this is a common procedure because the procedure of isolation of primary neural cells from adult animals is not optimized, a note of caution is needed. Another limitation of our study is the variety of neural cell types. It is a challenge to know the relative expression of receptors in projection neurons, in choline acetyltransferase (ChAT), parvalbumin (PV), calretinin (CR), or nitric oxide synthase interneurons and in astroglia and microglia, which may be at different degrees of activation depending on the disease status. Furthermore, receptor functionality in response to a given receptor ligand may vary from cell to cell [17] and at onset of disease when comparing naïve versus L-DOPA-treated individuals.

But in any case, pharmacological manipulation of RAS components presents potential in PD (Labandeira-Garcia et al., [29, 30])*.* One of the relevant findings in this paper is the insensitivity of AT_2_R to agonist treatment of striatal neurons. Indeed, those neurons express both Ang receptor types as previously demonstrated [19]. However, it is more remarkable that it becomes functional in the presence of candesartan. There are few examples of similar findings and a recent one consists of progressive decrease of adenosine A_2A_ receptor functionality upon coexpression of another adenosine receptor, A_2B_, and formation of A_2A_/A_2B_ heteroreceptor complexes. This phenomenon is due to allosteric inter-protomer interactions in the heteromer, i.e., the presence of one receptor blocks the signaling of the partner receptor in the complex [23]. Interestingly, the presence of an antagonist could make, as in the case of Ang receptor heteromers in striatal neurons, appear AT_2_R functionality back. Therefore, AT_1_R antagonists in these neurons may achieve two benefits, which are repressing the detrimental actions mediated by the AT_1_R while making that the AT_2_R becomes functional and provides the benefits associated to its activation. Furthermore, in terms of looking for interventions to prevent PD disease progression, there is enough information to agree that microglial cells may be key if there is a way to skew the physiology to acquire the M2 neuroprotective phenotype. On the one hand, neuronal alpha-synuclein produces an upregulation of AT_1_R while increasing in microglia the proportion of pro-inflammatory M1 versus neuroprotective M2 markers; accordingly, it is suggested that antagonists already used in hypertension and able to cross the blood-brain barrier may be repurposed for the therapy of PD [52]. On the other hand, microglial AT_2_Rs, constituent in AT_1/2_Hets, show promise as (i) they are upregulated in both parkinsonian conditions and in l-DOPA-induced dyskinesias and (ii) their activation is seemingly neuroprotective. Remarkably, AT_1/2_Hets do not show cross-antagonism, a property displayed by many heteromers and that would lead to a therapeutic dead end in terms of neuroprotection; instead, the antagonist of one receptor releases the brake on activation of the partner receptor. Taken together, the opposite action of AT_1_ and AT_2_ receptors, their expression in microglia, and the marked upregulation of AT_1/2_Hets lacking cross-antagonism but displaying antagonist-mediated cross-potentiation suggest that interventions aimed at antagonizing central AT_1_Rs to potentiate AT_2_R-mediated actions may be beneficial in PD. Further experimental effort is required to identify the cell types expressing the heteromer in the different parkinsonian conditions and to prove the functionality of the heteromer in in vivo conditions, something that at present is technically challenging.

## Conclusions

### Main conclusions

The present study demonstrates that AT_1_ and AT_2_ receptors form AT_1_/_2_Hets that are expressed in cells of the central nervous system. AT_1_/_2_Hets are novel functional units with particular signaling properties. Importantly, the coactivation of the two receptors in the heteromer reduces the signaling output of angiotensin. Remarkably, AT_1_/_2_Hets, which are expressed in both striatal neurons and microglia, show a cross-potentiation, i.e., candesartan, the antagonist of AT_1_ increases the effect of AT_2_ receptor agonists. In addition, the level of expression in the unilateral 6-OH-dopamine lesioned rat PD model increases upon l-DOPA treatment and is maximal in those animals that do not become dyskinetic.

### Importance and relevance of the study reported

These findings reported are potentially important because they indicate that boosting the action of neuroprotective AT_2_ receptors using an AT_1_ receptor antagonist constitutes a promising therapeutic strategy in PD. The strategy may consist of designing AT_1_R receptor antagonists able to readily cross the blood-brain barrier and effective in releasing the brake on the AT_2_ receptor. The fact that SARS-CoV-2 uses angiotensin-converting enzyme 2 (ACE2) as receptor, that ACE2 interacts with angiotensin receptors, that some COVID-19 patients with severe symptoms display a “cytokine storm” driven by macrophages and/or suffer neurological alterations, adds interest to the present work on the RAS system in microglia.

## Supplementary information

**Additional file 1.**

## Data Availability

All data are available upon request to the corresponding author(s).

## Abbreviations

6-hydroxy-DA: 6-Hydroxydopamine
AIMS: Abnormal involuntary movements
Ang: Angiotensin
Ang II: Angiotensin II
AT_1/2_Hets: AT1-AT2 Heterodimers
BRET: Bioluminescence resonance energy transfer
BSA: Bovine Serum Albumin
cAMP: Cyclic adenylic acid
CAN: Candesartan
CGP: CGP-42112A
ChAT: Choline acetyltransferase
CNS: Central nervous system
CR: Calretinin
Cy3: Cyano 3
DA: Dopamine
DIV: Days in vitro
DMEM: Dulbecco’s modified Eagle’s medium
DMR: Dynamic mass redistribution
DMSO: Dimethyl sulfoxide
FBS: Fetal bovine serum
FK: Forskolin
HBSS: Hanks’ balanced salt solution
HTRF: Homogeneous time resolved fluorescence
IFN-γ: Interferon-γ
l-DOPA: Levodopa
LPS: Lipopolysaccharide
MAPK: Mitogen-activated protein kinase
mBU: Milli bioluminescence resonance energy transfer units
NaCl: Sodium chloride
PBS: Phosphate-buffered saline
PD: Parkinson’s disease
PEI: Polyethylenimine
PKA: Protein kinase A
PLA: Proximity ligation assays
PV: Parvalbumin
RAS: Renin-angiotensin system
Rluc: Renilla luciferase
SERCA: Sarco/endoplasmic reticulum Ca^2+^-ATPase
SN: Substantia nigra
TR-FRET: Time-resolved fluorescence energy transfer
YFP: Yellow fluorescent protein
