# Supplementary material for: Angiotensin AT1 and AT2 receptor heteromer expression in the hemilesioned rat model of Parkinson’s disease that increases with levodopa-induced dyskinesia
Source: J Neuroinflammation. 2020 Aug 17;17:243. doi: 10.1186/s12974-020-01908-z (PMC7430099; doi:10.1186/s12974-020-01908-z)
Supplement: Supplementary file 1 — Additional file 1. [file 12974_2020_1908_MOESM1_ESM.docx]

Angiotensin AT_1_ and AT_2_ receptor heteromer expression in the hemilesioned rat model of Parkinson’s disease that increases with levodopa-induced dyskinesia

Rafael Rivas-Santisteban^1,2^, Ana I. Rodriguez-Perez^2,3^, Ana Muñoz^2,3^, Irene Reyes-Resina^1,2^, José Luis Labandeira-García^2,3^, Gemma Navarro^2,4,*^, Rafael Franco^2,5*^

Corresponding Authors:

- Rafael Franco

Dept. Biochemistry and Molecular Biomedicine. School of Biology. Prevosti Building. Universitat de Barcelona. Diagonal 643. 08028. Barcelona. Spain.

rfranco123@gmail.com

Tel +34934021213

- Gemma Navarro

Dept. Biochemistry and Physiology. School of Pharmacy and Food Sciences. Universitat de Barcelona. 08028. Barcelona. Spain.

g.navarro@ub.edu

Tel +34934034500

**Supplementary Material**

**
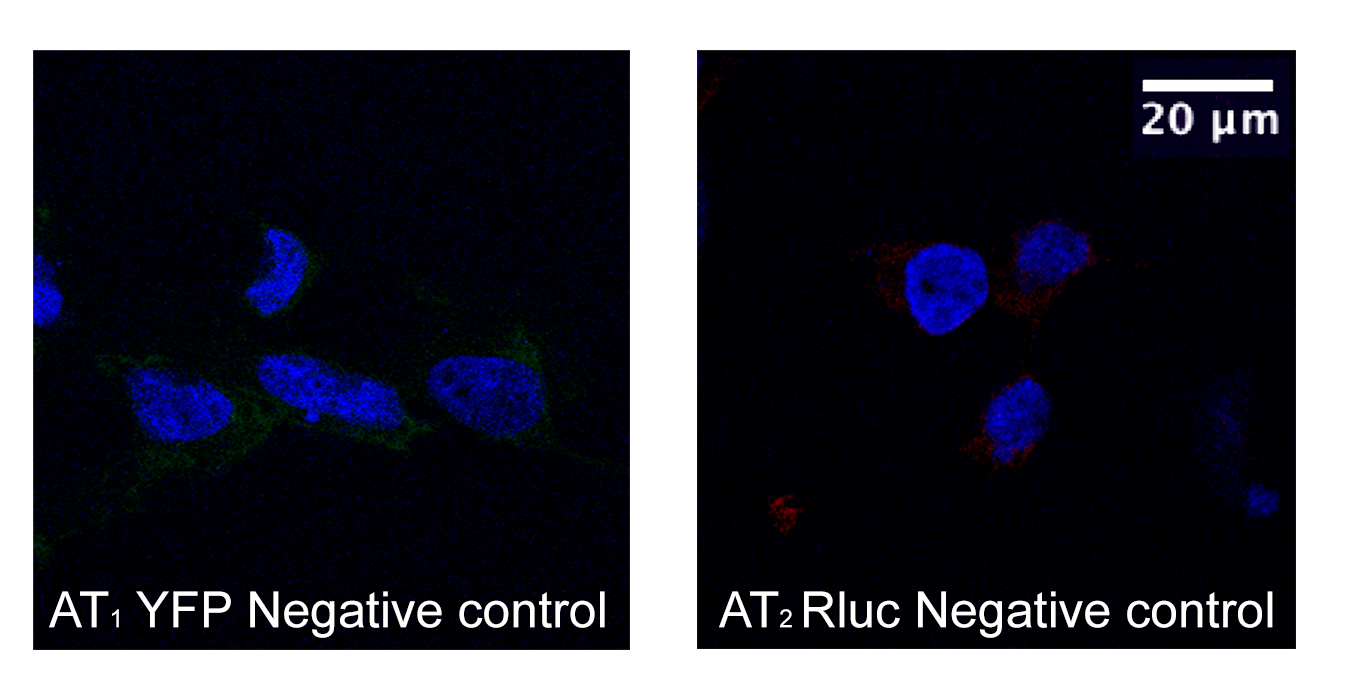
**

**Supplementary Figure S1. Immunocytochemistry negative controls.** Left: A negligible amount of green autofluorescence was detected in HEK-293T cells not expressing AT_2_-YFP. Right: Representative image of HEK-293T cells transfected with cDNA for AT_2_-RLuc (1 µg) processed for immunofluorescence staining using Cy3-conjugated secondary anti-mouse antibody but omitting the primary anti-RLuc antibody. Cell nuclei were stained with Hoechst (blue) in both cases. Scale bar: 20 µm.
